# Adherence patterns, risk factors and complications among patients with tuberculosis: a cross-sectional study at Nsawam Government Hospital

**DOI:** 10.1136/bmjph-2023-000618

**Published:** 2024-04-16

**Authors:** Abraham Norman Nortey, Amanda Adjoda, Amidu Alhassan, Godfred Yawson Scott

**Affiliations:** 1 University of Cape Coast, Cape Coast, Ghana; 2 Department of Midwifery, University of Health and Allied Sciences, Ho, Ghana; 3 Department of Nursing, University of Health and Allied Sciences, Ho, Ghana; 4 Department of Medical Diagnostics, Kwame Nkrumah University of Science and Technology, Kumasi, Ghana

**Keywords:** Public Health, Community Health, Infectious Disease Transmission, Vertical, Communicable Disease Control

## Abstract

**Background:**

Tuberculosis (TB) is the ninth leading cause of death worldwide, and the leading cause of death from a single infectious agent prior to COVID-19 pandemic. TB substantially affects adults during their most productive years. However, all age groups are at risk. More than 25% of cases and deaths occur in Africa. People infected with HIV are 20–30 times more likely to develop active TB. In this study, we determined the level of TB medication adherence, risk factors and complications of TB in outpatients attending the Nsawam Government Hospital.

**Method:**

This cross-sectional study randomly recruited 277 patients with TB attending the Nsawam Government Hospital for care. Structured questionnaires were used to collect sociodemographic information, adherence and lifestyle characteristics. Associations and multivariate logistic regression analysis were performed with 95% CIs. All p<0.05 were considered statistically significant.

**Results:**

Majority (36.1%) of the patients were between the ages of 29 and 38. Majority (63.9%) of them had high adherence to TB medication. Participants earning less than 500 Ghana Cedis (Ghc) (adjusted OR (aOR)=8.85. 95% CI (1.59 to 49.24), p=0.013), patients with TB who indicated having complication (joint pain, spinal pain, heart disorders, liver or kidney problems) as a result of TB (aOR=2.81, 95% CI (1.58 to 4.99), p<0.001), respondents who mentioned living with people infected with TB (aOR=3.23. 95% CI (1.24 to 8.37), p=0.016) were the independent predictors of adherence to TB medication.

**Conclusion:**

The study findings revealed that participants exhibited commendable adherence to TB medication. Notably, adherence levels were found to be linked to several factors, including income, medication side effects and cohabitation with individuals infected with TB. The positive trend in adherence underscores the importance of considering socioeconomic factors, as lower income was identified as a potential barrier to consistent medication adherence

**WHAT IS ALREADY KNOWN ON THIS TOPIC**

**WHAT THIS STUDY ADDS**

- The study provides insight into the level of medication adherence among patients with TB at Nsawam Government Hospital in Ghana.
- The research highlights various factors that influence medication adherence among patients with TB in this specific context, including income levels, medication side effects and living with TB-infected individuals.

**HOW THIS STUDY MIGHT AFFECT RESEARCH, PRACTICE OR POLICY**

- Early recognition and management of side effects and discomfort can enhance patient well-being and adherence to medication.
- Income levels emerged as a significant factor influencing adherence. This highlights the importance of addressing socioeconomic barriers to TB treatment in low-income populations.

## Introduction

 Tuberculosis (TB) remains a major public health challenge globally, with approximately 10 million new cases and 1.4 million deaths reported annually.^1 2^ Africa is the second highest TB endemic region in the world and contributes to about 25% of global TB incident cases.^3^ In Ghana, TB is a significant health problem, with an estimated incidence rate of 126 cases per 100 000 population.^2^ Prior to the global outbreak of the COVID-19 pandemic, TB was the leading cause of death from a single infectious agent.^2^

TB is caused by a bacterium called *Mycobacterium tuberculosis*. Although the bacteria usually attack the lungs, they can also affect the kidney, spine and brain.^4^ Untreated TB carries a mortality rate of more than 50%. More importantly, it can lead to devastating complications.^5^ The most terrifying concern is that most middle-aged adults in underdeveloped nations who develop TB die prematurely than in developed countries due to poor adherence to medication and poor management.^6^

According to the WHO, adherence refers to the extent to which a person’s behaviour, taking medication, following a diet and/or executing lifestyle changes correspond with agreed recommendations from a healthcare provider.^7^ Patients must take the anti-TB medications for at least six uninterrupted months in order to be effective with their TB therapy.^8^ However, almost half of all patients with TB worldwide are unable to complete the prescribed treatment course, indicating that the other half do not adhere to TB treatment.^9^ A false impression of recovery before completing therapy, drug side effects, ignorance as well as depression, forgetfulness and failure to obtain medications from the medical facility are some variables that have been proposed to contribute to non-adherence to TB treatment.^8 9^ Other variables that may contribute to non-adherence include economic hardships, regional challenges, and a preference for conventional therapy.^9^

The delivery system of TB medication in Ghana has undergone significant developments since the preindependence era. In 1994, Ghana adopted the WHO Directly Observed Treatment Shortcourse (DOTS) Strategy, emphasising political commitment, sputum smear microscopy diagnosis, standardised supervised treatment, uninterrupted drug supply and robust recording and reporting. DOTS was implemented nationwide, achieving 100% coverage by WHO standards in 2000. Presently, the National TB Control Programme (NTP) focuses on maintaining and expanding DOTS, ensuring regular drug supply, surveillance and capacity building.^10^ Typically, patients with TB in Ghana are treated on an outpatient basis, with DOTS involving direct observation to enhance adherence.

In patients with communicable conditions, such as TB, adherence to medication is a critical aspect of the successful treatment and prevention of drug resistance. Despite the availability of effective medications to treat TB, non-compliance with medication remains a major obstacle to achieving successful treatment outcomes among patients. The problem of non-compliance to medication among patients with TB is particularly prevalent in low and middle-income countries, including Ghana, where poverty, inadequate health infrastructure and limited access to healthcare services contribute to poor treatment adherence.^2^ In this study, we determined the level of medication adherence, risk factors and complications of TB in patients attending the Nsawam Government Hospital, Ghana.

## Materials and methods

### Study design

This hospital‐based cross‐sectional study was conducted between August 2022 and July 2023 at Nsawam Government Hospital after obtaining permission from the Institutional Ethics Committee.

### Study site

Nsawam Government Hospital is well known in the Eastern Region to be one of the best in the areas of Quality Assurance and Infection Prevention. It is located at Nsawam Adoagyiri Municipality and serves an estimated population of approximately 110 677. Nsawam Government Hospital has 135 bed capacity. The selection of Nsawam Government Hospital as the study site is justified based on its strong reputation for quality healthcare services suggesting that it maintains high standards in patient care and management, strategic location within the Eastern Region, which allows for the inclusion of a diverse and representative population in the study. The hospital’s capacity to accommodate a considerable number of patients further supports its suitability for conducting a thorough cross-sectional study on adherence patterns, risk factors and complications among patients with TB.

### Inclusion and exclusion criteria

This study included the following participants: those who have been diagnosed with TB and are 18 years and above, those who started TB treatment at the hospital and have been on medication for at least 2 weeks. The study excluded participants who were disabled, severely ill, participants with underlying medical conditions that may affect their compliance with treatment, participants who were pregnant or breast feeding. Participants who did not give formal consent to participate in the study were also excluded.

### Sample size estimation

To address potential sampling bias, we randomly recruited a total of 277 participants after the aim of the study had been clearly explained to them. The sample size was estimated using the formula$n=\frac{Z^{2}\;Pq}{d²}$

Using a prevalence of 22.9% obtained from a similar study.^11^ The estimated sample size (N) was 271. To increase statistical power and account for non-response bias, 277 TB outpatients were sampled for the study.

### Data collection

A well‐structured questionnaire was developed by reviewing relevant journals. During the design phase of this research, we actively sought input and feedback from patients who have been diagnosed with TB and members of the public with an interest in public health. We conducted pilot interviews and discussions with a group of 20 patients with TB to ensure the clarity and relevance of the research questionnaire. Their insights helped us refine the questionnaire and make it more patient-friendly, ensuring that it addressed relevant concerns and was easy to understand. Throughout the conduct phase of the study, we maintained open channels of communication with patients with TB attending the Nsawam Government Hospital. Their feedback and experiences provided valuable context for understanding the challenges and realities of TB treatment adherence, lifestyle factors and complications. This information was instrumental in shaping our research approach and interpretation of the findings. It is important to note that while patients and the public were actively involved in the design and conduct phases of this research, they were not involved as formal research participants.

Simple Randomized Sampling without Replacement was employed. The questions were asked through face‐to‐face interviews with the patients. The questionnaire was originally in English but was carefully translated into the local language of the study population during the interviews. The participants’ responses were then translated back into English while maintaining their intended meaning. The questionnaire was structured in four sections. The first section was used to obtain sociodemographic data such as age, sex, ethnicity, education, marital status, number of children, religion, occupation, type of employment, monthly income and duration of residency in their current location. The second section included questions on current use of TB medication, smoking habits, alcohol use, exercise frequency, living or working in residential care facilities or healthcare settings, exposure to individuals infected with TB and dietary habits. The third section included possible complications of TB. Finally, the fourth section was dedicated to exploring aspects related to TB medication and adherence. Morisky Medication Adherence Scale (MMAS) was used to access adherence to TB medications.

The MMAS serves as a self-reported assessment instrument designed to assess medication adherence across diverse patient populations. The MMAS is specifically employed for screening non-adherence in individuals with chronic conditions. This tool is recognised for its simplicity, cost-effectiveness and ease of use in evaluating medication compliance. In this study, adherence level was calculated using eight set of items. All the items had options, which ranged from ‘never to all the time’. Being a Likert scale, the items were recoded such that, never had a code of 0, sometimes had a code of 1, most of the times had a code of 2 and all the time had a code of 3. Individual scores were consolidated by summation, resulting in a total score for each participant. The median value, calculated as 12, served as the cut-off point for categorising adherence levels. Scores below the median were classified as indicating low adherence, while those at or above the median were considered reflective of high adherence.

### Data analysis

Data entry was done using Microsoft Excel V.2016 and analysis was performed using STATA Windows V.17.0 and Epi Data V.4.1. Categorical data were presented as frequency (proportion). The normality of continuous variables was tested by Kolmogorov-Smirnov. Non-parametric data were presented as median (IQR). Differences between groups were tested for significance by Mann-Whitney U‐test for non-parametric data (for continuous variables) and χ^2^ or Fisher exact test was used to determine the association of sociodemographic characteristics, including age categories, gender, marital status and adherence level among the participants. Multivariate logistic regression analysis was performed to compare the OR of sociodemographic and lifestyle characteristics of the study participants. All statistical results obtained were considered at a significant value of p<0.05.

## Results

### Sociodemographic characteristics of respondents

Table 1 shows the sociodemographic characteristics of the study participants. Majority (36.1%) of the patients were between the ages of 29 to 38 and more than half (63.9%) were women. About half (51.3%) of the participants had secondary level of education. More than half (53.8%) of the participants were married. Most (69%) of the participants were self-employed. The number of children that patient had varied, with the majority (64.3%) having 1–4 children. Average income of patients varied, with majority (40.1%) earning between 500 and 999 Ghc, majority have stayed at their abode between 5 and 10 years (47.3%) (table 1).

**Table 1:** Sociodemographic characteristics of respondents

Variable	Frequency (n=277)	Percentage
Age mean (±SD)	37.6 (±11.2)	
18–28	58	20.9
29–38	100	36.1
39–48	81	29.2
49+	38	13.8
Sex		
Male	100	36.1
Female	177	63.9
Ethnicity		
Akan	155	56.0
Ewe	69	24.9
Guan	10	3.6
Others	43	15.5
Educational level		
No formal education	21	7.6
Basic level	83	30.0
Secondary level	142	51.3
Tertiary level	31	11.1
Marital status		
Single	79	28.5
Married	149	53.8
Divorced/separated	40	14.4
Widowed	9	3.3
Number of children		
None	55	19.9
1– 4	178	64.3
5 and above	44	15.8
Religion		
Christianity	241	87.0
Islam	36	13.0
Occupation		
Unemployed	79	28.5
Civil servant	7	2.5
Self-employed	191	69.0
Type of employment	**N=198**	
Informal sector	191	96.5
Public servant	7	3.5
Average income (monthly)		
Not working/dependent	72	26.0
<500 Ghc	39	14.1
500–999 Ghc	111	40.1
1000–1500 Ghc	49	17.7
Above 1500 Ghc	6	2.2
Duration of stay at abode	10.4 (7.0)	
< 5 years	108	39.0
5–10 years	131	47.3
11+years	38	13.7

Data areis presented as frequency (%).

### Lifestyle risk factors among patients with TB

**Figure 1:**
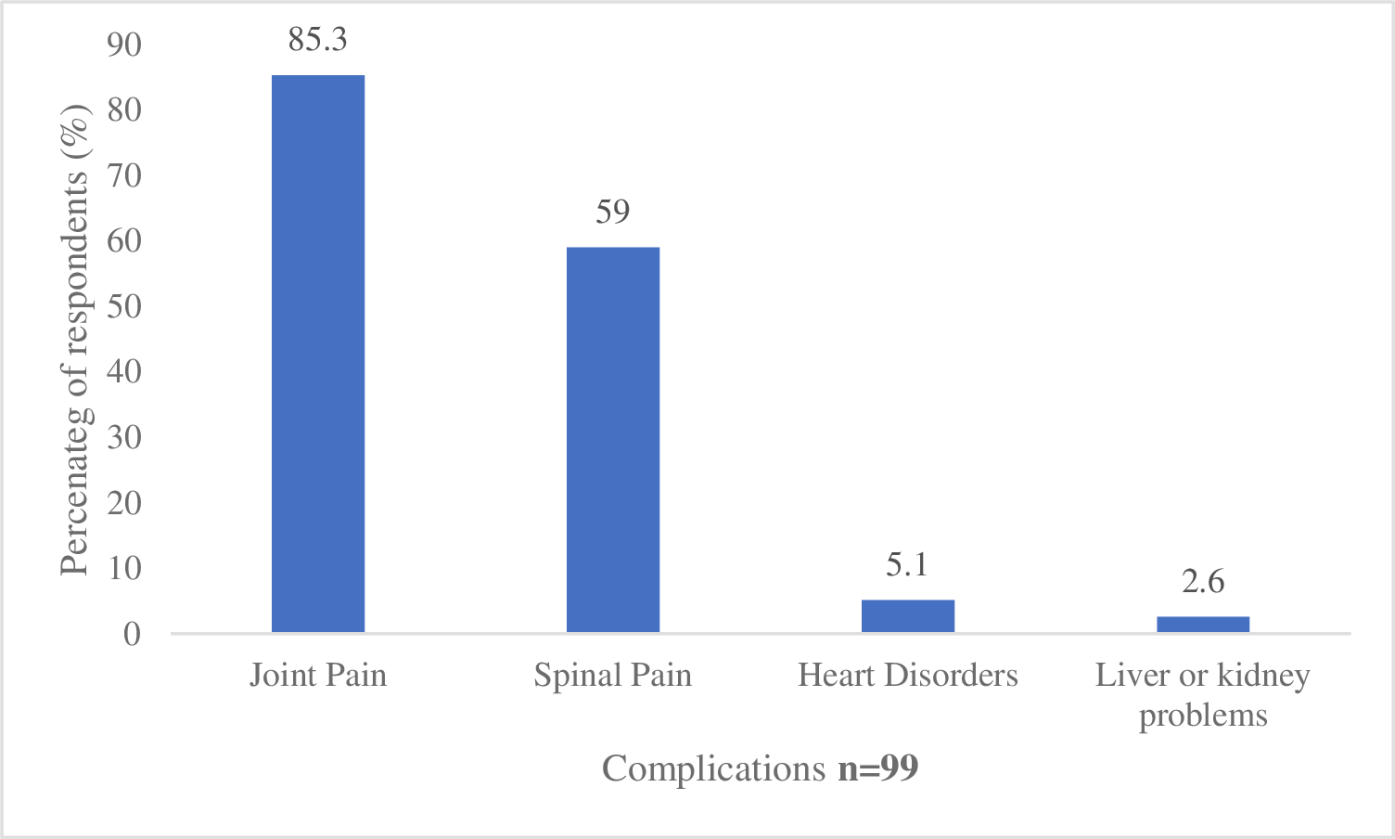
Complications due to TB. TB, tuberculosis.

**Figure 2:**
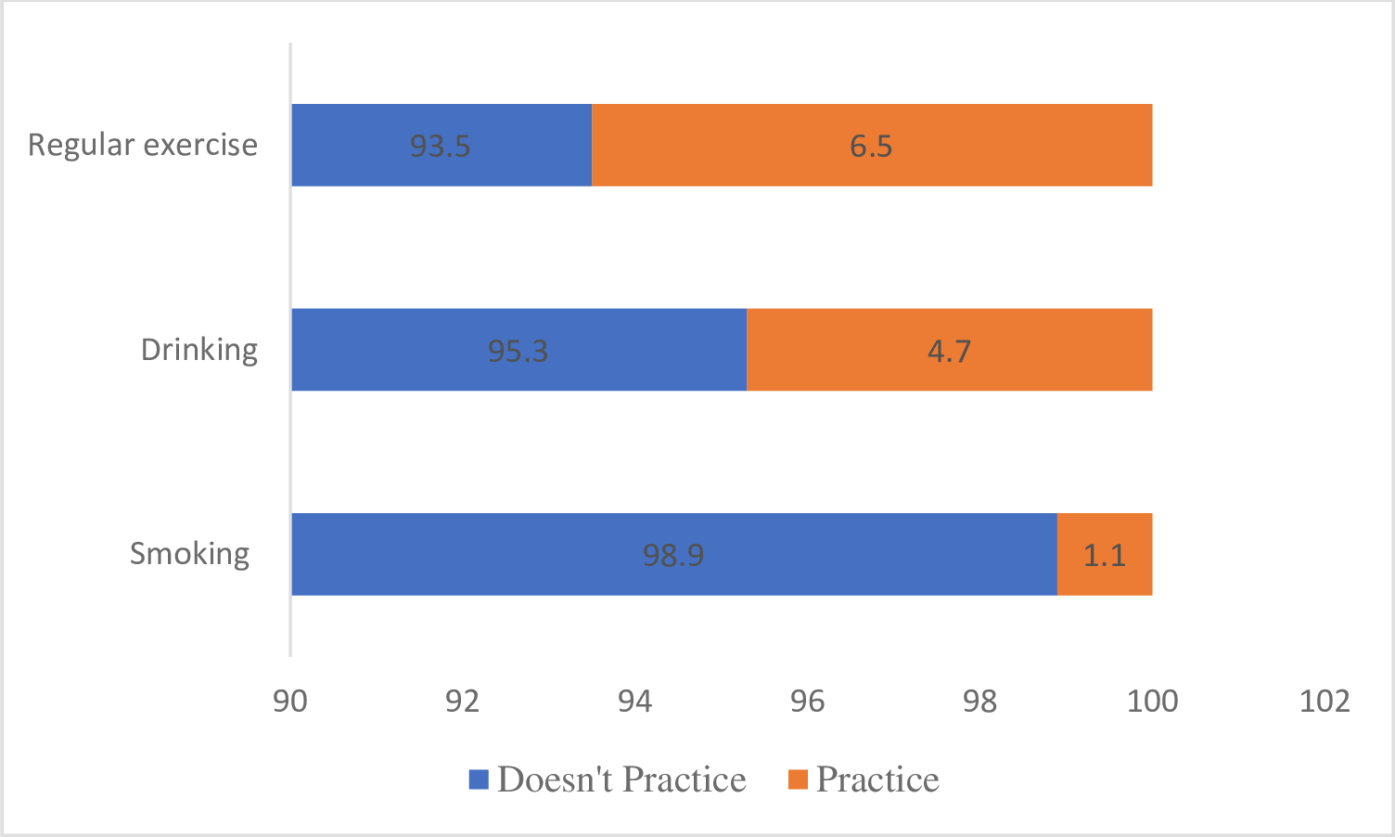
Stack graph of different habitual status.

Table 2 shows the lifestyle risk factors among the study participants. Concerning the use of TB medication, 69.7% of the patients responded that they currently use TB medication. Most (98.9%) of the patients indicated that they do not smoke. Majority (66.7%) of those who smoke have smoked for 1–2 years, and all of them smoke less than five cigarettes a day on average. Most (95.3%) of the patients do not take alcohol, while only 4.7% of them do. More than half (53.8%) of those who take alcohol have taken it for more than 5 years. Most (93.5%) of the participants do not exercise regularly, while 6.5% of them do. Of those who exercise, about half (50%) do it for an hour a day. Majority (93.5%) of the participants do not live or work in a residential care facility. Few (3.2%) of them work in healthcare. Few (8.3%) live with someone infected with TB. Concerning diet, majority (78.7%) of the patients eat grains and cereals sometimes. Also, 60.3% of the patients eat protein sometimes, while 39.0% eat it often, and only 0.7% never eat it. Less than half (36.7%) of the participants reported having complications as a result of TB (table 2). Figure 1 shows the complications reported by the study participants. Majority (85.3%) of them reported joint pain as the complication due to TB. Figure 2 shows a stack graph of different habitual status of the study participants.

**Table 2:** Lifestyle risk factors

Variable	Frequency (n=277)	Percentage
Do you currently use any form of tuberculosis medication?		
No	84	30.3
Yes	193	69.7
Do you smoke tobacco?		
No	274	98.9
Yes	3	1.1
Number of years of smoking	**N=3**	
1–2	2	66.7
3	1	33.3
Average cigarettes smoke		
<5	2	66.7
5+	1	33.3
Do you take alcohol?		
No	264	95.3
Yes	13	4.7
Number of years of drinking	**N=13**	
<5	6	46.2
5+	7	53.8
How often you take alcohol?		
<5 times	8	61.5
5+times	5	38.5
Do you exercise regularly?		
No	259	93.5
Yes	18	6.5
Duration of exercise	**N=18**	
< 1 hour	5	27.8
1 hour	9	50.0
> 1 hour	4	22.2
How often do you exercise?		
Everyday	6	33.3
Once a week	2	11.1
Twice and above	10	55.6
Living or working in a residential care facility		
No	259	93.5
Yes	18	6.5
Healthcare worker		
No	268	96.8
Yes	9	3.2
Living with someone infected with TB		
No	254	91.7
Yes	23	8.3
How often do you eat grains and cereals?		
Never	6	2.2
Often	53	19.1
Sometimes	218	78.7
How often do you eat protein?		
Never	2	0.7
Often	108	39.0
Sometimes	167	60.3
Do you have complications as a result of tuberculosis?		
No	178	64.3
Yes	99	36.7

Data areis presented as frequency (%).

### Adherence level towards TB medication

**Figure 3:**
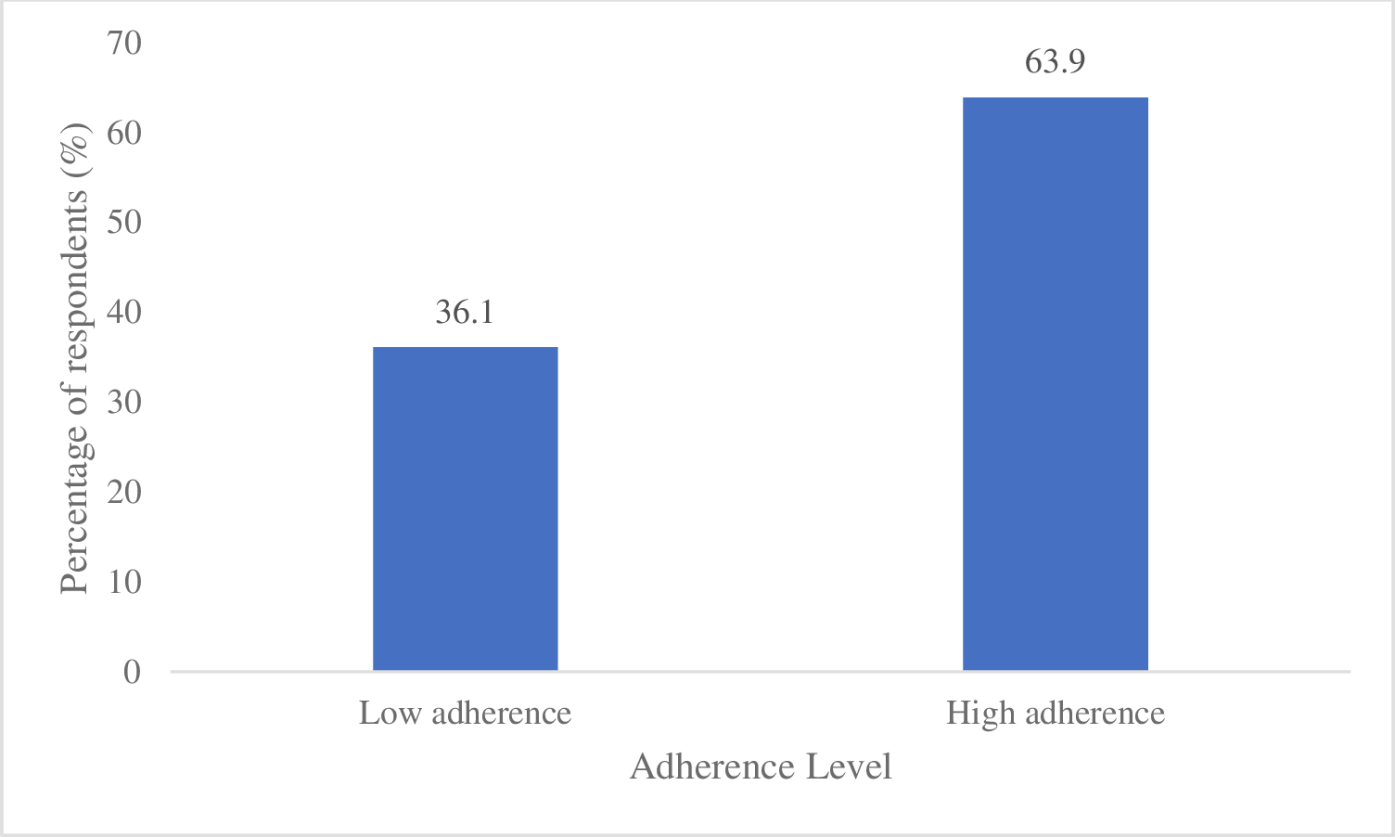
Adherence level towards TB medication. TB, tuberculosis.

Table 3 shows the adherence level of the participants towards TB medication. Majority (42.6%) of patients reported taking medication most of the time. About 6.9% of the patients never took the medication for TB as prescribed by the doctor. Most (63.9%) of the patients reported that they sometimes have problems remembering to take their medication. Only 4.3% of the patients reported that they have problems remembering to take their medication. Although majority (50.2%) of the patients reported taking all their TB doses as instructed most of the time or all the time, 2.2% reported never taking all their doses as instructed. Majority (59.9%) reported that they sometimes skip their medications due to various reasons. Most (61.0%) of them reported that they use the medication most of the time because it makes them feel better. Although majority (34.7%) of them reported receiving social support from family and friends most of the time to take their medications, 17.3% of them reported that they never receive social support from family and friends to take their mediation. Majority (39.8%) of them reported that they sometimes miss their clinic visits when scheduled, which is also a cause for concern. Most (75.8%) of them reported that they sometimes feel uncomfortable about adhering to their medication plan, and 18.1% of patients indicated they never felt uncomfortable about adhering to their medication (table 3). Figure 3 shows the adherence levels of the study participants. Majority (63.9%) of them had high adherence to TB medication.

**Table 3:** Adherence level towards tuberculosis medication

Variable	Nevern (%)	Sometimes n (%)	Most of the times n (%)	All the timen (%)
Do you take medication for your TB as prescribed by the doctor?	19 (6.9)	31 (11.2)	118 (42.6)	109 (39.3)
Do you have problems remembering to take your medication?	61 (22.0)	177 (63.9)	27 (9.8)	12 (4.3)
I take all my TB doses as instructed by the doctor/nurse	6 (2.2)	37 (13.4)	139 (50.2)	95 (34.3)
I skip my medications due to some reasons such as during.	81 (29.2)	166 (59.9)	27 (9.8)	3 (1.1)
I use the medication because it makes me feel better.	15 (5.4)	19 (6.9)	169 (61.0)	74 (26.7)
Do you receive social support from family and friends to take your medications?	48 (17.3)	68 (24.6)	96 (34.7)	65 (23.4)
I miss clinic visit when scheduled	94 (33.9)	110 (39.8)	33 (11.9)	40 (14.4)
Have you ever felt uncomfortable about adhering to your medication plan?	50 (18.1)	141 (50.9)	69 (24.9)	17 (6.1)

Data areis presented as frequency (%).

TBtuberculosis

### Associations, univariate and multivariate logistic regression model of sociodemographic and lifestyle risk predictors of adherence among study participants

Table 4 shows the association between sociodemographic characteristics, lifestyle risk factors and adherence level of study participants. There was a significant association between occupation (p=0.023), average income (p=0.023), duration of stay at abode (p=0.020), smoking tobacco (p=0.022), living or working in a residential healthcare facility (p=0.002), healthcare worker (p=0.012), living with someone infected with TB (p=0.013), often eat grains and cereals (p=0.036), have complications as a result of TB (p=0.002) and level of adherence. On the contrary, there was no significant association between age (p=0.457), sex (p=0.450), ethnicity (p=0.259), educational level (p=0.141), marital status (p=0.619), number of children (p=0.841), religion (p=0.999), sex (p=0.450), often eat protein (p=0.696) and level of adherence.

**Table 4:** Association between sociodemographic characteristics, lifestyle risk factors and adherence level of study participants

Variable	Adherence level		X^2^(P value)/Fisher’s exact
	Low n (%)	High n (%)	
Age			2.604 (0.457)
18–28	16 (27.6)	42 (72.4)	
29–38	38 (30.0)	62 (62.0)	
39–48	30 (37.0)	51 (63.0)	
49+	16 (42.1)	22 (57.9)	
Sex			0.570 (0.450)
Male	39 (39.0)	61 (61.0)	
Female	61 (34.5)	116 (65.5)	
Ethnicity			0.259
Akan	50 (32.3)	105 (67.7)	
Ewe	27 (39.1)	42 (60.9)	
Guan	6 (60.0)	4 (40.0)	
Others	17 (39.5)	26 (60.5)	
Educational level			5.463 (0.141)
No formal education	9 (57.1)	12 (57.1)	
Basic level	35 (42.2)	48 (57.8)	
Secondary level	42 (29.6)	100 (70.4)	
Tertiary level	14 (45.2)	17 (54.8)	
Marital status			0.619
Single	26 (32.9)	53 (67.1)	
Married	53 (35.6)	96 (64.4)	
Divorced/separated	18 (45.0)	22 (55.0)	
Widowed	3 (33.3)	6 (66.7)	
Number of children			0.346 (0.841)
None	18 (32.7)	37 (67.3)	
1–4	66 (37.1)	112 (62.9)	
5 and above	16 (36.4)	28 (63.6)	
Religion			0.00 (0.999)
Christianity	87 (36.1)	154 (63.9)	
Islam	13 (36.1)	23 (63.9)	
Occupation			**0.023**
Unemployed	29 (36.7)	50 (63.3)	
Civil servant	6 (85.7)	1 (14.3)	
Self-employed	65 (34.0)	126 (66.0)	
Average income (monthly)			
Not working/dependent	24 (33.3)	48 (66.7)	**0.023**
<500 Ghc	22 (56.4)	17 (43.6)	
500–999 Ghc	36 (32.4)	75 (67.6)	
1000–1500 Ghc	14 (28.6)	35 (71.4)	
Above 1500 Ghc	4 (66.7)	2 (33.3)	
Duration of stay at abode			**7.808** (0.020)
< 5 years	39 (36.1)	69 (63.9)	
5–10 years	40 (30.5)	91 (69.5)	
11+years	21 (55.3)	17 (44.7)	
Do you smoke tobacco?			**5.220** (0.022)
No	92 (34.9)	172 (65.2)	
Yes	8 (61.5)	5 (38.5)	
Living or working in a residential care facility			**0.002**
No	87 (33.6)	172 (66.4)	
Yes	13 (72.2)	5 (27.8)	
Healthcare worker			**0.012**
No	93 (34.7)	175 (65.3)	
Yes	7 (77.8)	2 (22.2)	
Living with someone infected with TB			**6.671** (0.013)
No	86 (33.9)	168 (66.1)	
Yes	14 (60.9)	9 (39.1)	
How often do you eat grains and cereals?			**0.036**
Never	0 (0.0)	6 (100.0)	
Often	25 (47.2)	28 (52.8)	
Sometimes	75 (34.4)	143 (65.6)	
How often do you eat protein?			0.696
Never	0 (0.0)	2 (100.0)	
Often	38 (35.2)	70 (64.8)	
Sometimes	62 (37.1)	105 (62.9)	
Do you have complications as a result of TB?			**10.242** (0.002)
No	52 (29.2)	126 (70.8)	
Yes	48 (48.5)	51 (51.5)	

Data areis presented as frequency (%); Chiχ2 square/ Fisher’s Exact, p- value ˂0.05 was considered statistically significant for adherence level. The bold values indicate p- values, which are statistically significant.

TBtuberculosis

Table 5 shows the binary logistic regression between adherence level and its associated factors. In a univariate logistic regression model, civil servants (cOR=7.41, 95% CI (1.19 to 46.34), p=0.032) were seven times more likely to adhere to TB medication compared with unemployed participants. Participants earning less than 500 Ghc (cOR=2.55, 95% CI (1.15 to 5.61), p=0.021) were almost three times more likely to adhere to TB medication compared with participants who were not working/dependent. Participants who smoke tobacco (cOR=2.29, 95% CI (1.12 to 7.57), p=0.028) were two times more likely to adhere to TB medication compared with participants who do not smoke tobacco. Participants who live or work in a residential care facility (cOR=4.84, 95% CI (1.73 to 13.48), p=0.003) were almost five times more likely to adhere to TB medication compared with participants who do not live or work in a residential care facility. Healthcare workers (cOR=5.63. 95% CI (1.32 to 24.08), p=0.020) were almost six times more likely to adhere to TB medication compared with non-healthcare workers. Participants living with someone infected with TB (cOR=2.97, 95% CI (1.26 to 7.01), p=0.013) were almost three times more likely to adhere to TB medication compared with participants who are not living with someone infected with TB. Participants who have complications as a result of TB (cOR=2.27, 95% CI (1.37 to 3.77), p=0.002) were three times more likely to adhere to TB medication compared with participants who do not have complications as a result of TB.

**Table 5:** Univariate and multivariate logistic regression model of sociodemographic characteristics and lifestyle risk predictors of adherence

Variable	cOR (95%, CI), P value	aOR (95%, CI), P value
Occupation		
Unemployed	1	1
Civil servant	**7.41 (1.19 to 46.34), 0.032**	3.83 (0.11 to 135.36), 0.461
Self-employed	0.89 (0.51 to 1.53), 0.664	0.26 (0.05 to 1.34), 0.109
Average income (monthly)		
Not working/dependent	1	1
<500 Ghc	**2.55 (1.15 to 5.61), 0.021**	**8.85 (1.59 to 49.24), 0.013**
500–999 Ghc	0.96 (0.51 to 1.79), 0.891	3.94 (0.72 to 21.45), 0.113
1000–1500 Ghc	0.81 (0.37 to 1.76), 0.594	3.11 (0.49 to 19.50), 0.227
Above 1500 Ghc	3.56 (0.74 to 18.01), 0.124	0.73 (0.01 to 46.38), 0.880
Duration of stay at abode		
< 5 years	1	1
5–10 years	0.78 (0.45 to 1.33), 0.362	0.65 (0.35 to 1.19), 0.161
11+ years	**2.16 (1.03 to 4.54), 0.042**	1.81 (0.82 to 4.01), 0.145
Do you smoke tobacco?		
No	1	1
Yes	**2.92 (1.12 to 7.59), 0.028**	1.87 (0.61 to 5.73), 0.273
Living or working in a residential care facility		
No	1	1
Yes	**4.84 (1.73 to 13.48), 0.003**	2.57 (0.75 to 8.87), 0.135
Healthcare worker		
No	1	1
Yes	**5.63 (1.32 to 24.08), 0.020**	2.86 (0.56 to 14.71), 0.209
Living with someone infected with TB		
No	1	1
Yes	**2.97 (1.26 to 7.01), 0.013**	**3.23 (1.24 to 8.37), 0.016**
How often do you eat grains and cereals?		
Never	1	
Often	11.63 (0.63 to 216.89), 0.100	
Sometimes	6.84 (0.38 to 123.06), 0.192	
Do you have complications as a result of TB?		
No	1	1
Yes	**2.27 (1.37 to 3.77), 0.002**	**2.81 (1.58 to 4.99), <0.001**

Multivariate logistic regression analysis was performed to obtain odd ratios. P- value of<0.05 was considered statistically significant. The bold values indicate p- values, which are statistically significant.

1.00referenceaORadjusted ORcORcrude ORTBtuberculosis

After adjusting for possible cofounders in the multivariate logistic regression model, participants earning less than 500 Ghc (aOR=8.85. 95% CI (1.59 to 49.24), p=0.013), patients with TB who indicated having complication as a result of TB (aOR=2.81. 95% CI (1.58 to 4.99), p<0.001), respondents who mentioned living with people infected with TB (aOR=3.23, 95% CI (1.24 to 8.37), p=0.016) were the independent predictors of adherence to TB medication (table 5).

## Discussion

### Lifestyle risk factors

Majority (69.7%) of the patients said they were currently taking TB medication, which is essential for managing and treating the condition, but 30.3% of the patients said they were not taking their TB medicine, which would mean they were not following their treatment plan. To ensure improved treatment outcomes, it is crucial to investigate the causes of non-compliance and address them as soon as possible. Only 1.1% of the patients admitted to smoking, a remarkably low number when compared with the overall population. However, the study found that 66.7% of smokers have been doing so for between 1 and 2 years, which may point to a more recent start to the habit. This, however, is still a cause for concern. Comparably, smoking has been shown to be one of the risk factors that impairs the host’s defence system against TB infection and diseases WHO,^12^ Quan *et al*^13^ and a multicentre study conducted in Malaysia by Khan *et al*.^14^ Smoking weakens the lungs and raises the risk of respiratory infections; thus, it may still be dangerous for people with TB.

Regarding alcohol consumption, 4.7% of the patients reported drinking, with more than half of them drinking more than five times a year. Although the percentage of alcohol consumption among patients with TB is relatively low, it is crucial to educate patients on the potential negative effects of alcohol on their treatment outcomes. Contrastingly, a study conducted among 234 patients with TB on examining the social status, risk factors and lifestyle changes of patients with TB in Sri Lanka during the treatment period highlighted alcohol consumption as one of the risk factors among 27.4% of the patients with TB.^15^ The discrepancy in the study may be attributed to the study setting. Irrespective of differences in proportion in the studies, alcohol consumption may also weaken the immune system and increase the risk of developing other infections.

The vast majority of patients (93.5%) said they did not exercise frequently. Exercise has been demonstrated to improve lung function and lower the chance of developing chronic diseases, among other health advantages. Exercise as a risk factor among patients with TB is similarly found in a study undertaken on Lifestyle Risk Factors Associated with Tuberculosis Patients in Asir Region of Saudi Arabia.^16^ TB patients’ general health and well-being may be enhanced by encouraging and promoting frequent exercise. It is, hence, crucial for health workers to emphasise the need for patients with TB regularly engage themselves in physical activity.

Moreover, 36.7% of the patients said that TB problems had affected them. This can point to the necessity for more vigorous therapy or oversight. To stop more complications from arising and to enhance the effectiveness of treatment, it is critical to recognise and handle complications as soon as they arise.

### Adherence level towards TB medication

TB is a global health problem and just like the treatment and management of any disease, medication adherence is critical to the treatment of TB. In general, in this study, there was high adherence level (63.9%). In comparison to women, non-adherence was high among men, which could be linked to men’s health-seeking behaviour. For instance, men may prioritise their work and family responsibilities over their own health, leading them to neglect seeking timely and consistent treatment for TB. According to the current study, the majority of patients (42.6%) and 39.3% said they took their medication as directed by their doctor most of the time and always, respectively, which points to a relatively high level of medication adherence among patients with TB at Ghana’s Nsawam Government Hospital. This finding parallels the results from a study on the level of attitude, medication adherence and quality of life among patients with Tuberculosis in Topoyo Public Health Center, Central Mamuju Regency, Indonesia.^17^ The study established that 45.7% of the study participants showed moderate level of attitude in medication adherence. Owing to the fact that adherence is essential to TB treatment, it is paramount to encourage and intensify education for patients with TB for effective results.

In addition, most (63.9%) of the patients reported that they only have problems sometimes remembering taking their medication. Patient’s age, level of education and health status may play a role in their ability to remember to take their medication regularly as older patients may have memory issues in remembering, while those with lower levels of education may have difficulty understanding the importance of adherence. This demonstrates the necessity for medical professionals to create methods to aid patients in remembering to take their medication, such as by setting reminders. Interestingly, close to one-fourth (22.0%) mentioned that they never have problems remembering their medication intake. This finding could possibly be ascribed to social support and access to healthcare services as these may contribute to medication adherence. Patients with a strong social support system and access to healthcare may be more likely to adhere to their medication regimen while patients who lack social support or face barriers to accessing healthcare may struggle with adherence.

In terms of social support, the study discovered that while 34.7% and 23.4% of patients said they got it all the time or most of the time to take their medications, respectively, 24.6% and 17.3% of patients said they only got it occasionally or never. Patients who receive social support from family, friends and healthcare providers are more likely to adhere to their medication regimen and complete the treatment. This finding agrees with a systematic review study on the role of social support on treatment adherence in patients with TB.^18^ The study established that social support that includes family support, peer support and support from health workers is one of the driving factors for treatment adherence in patients with TB. This emphasises how crucial it is to include family members or other caregivers in the patient’s care and treatment plan to support and encourage medication adherence. Social support can take many forms, such as reminders to take medication, help with transportation to healthcare appointments, emotional support and assistance with daily tasks.

A significant number of patients (73.7%) reported irregularly skipping scheduled clinic sessions, according to the study. This is also a problem because it is critical for patients to visit the clinic frequently, so that their progress can be tracked and their treatment plan can be modified as necessary. Healthcare professionals ought to engage with patients to resolve any obstacles to attending clinic sessions and stress the significance of regular attendance

### Factors associated with non-compliance to medication among patients living with TB

The factors that were found to be significantly associated with the adherence level towards TB included medication, average income, duration of stay at the current place of residence, smoking status, living or working in a residential care facility, working in healthcare, living with someone infected with TB, how often individuals eat grains and cereals and presence of complications. However, in adjusting for the OR, individuals who earned less than 500 GH were eight times more likely to have low adherence towards TB medication compared with those who were not working. One possible reason for this could be financial constraints as this may lead to difficulties in accessing medication, affording transportation to health facilities or meeting other healthcare-related costs. This finding aligns with results from a study on factors related to treatment adherence in patients with TB in Pereira, Colombia where economic impact was an important factor in medication non-adherence.^19^ Consequently, these individuals may struggle to adhere to the prescribed medication regimen.

Furthermore, patients with TB who experienced complications as a result of the disease were more likely to have low adherence to medication. This finding suggests that the presence of complications may negatively affect a patient’s ability or willingness to adhere to the treatment. This is similar to findings from a systematic review study on patient adherence to TB treatment by Munro *et al*.^20^ The study found that side effects were linked to low adherence to TB treatment. Thus, complications can cause discomfort, pain or other adverse effects, which might discourage patients from following their medication regimen strictly.

In addition, respondents who mentioned living with people infected with TB were three times more likely to have low adherence to TB medication compared with those who were not living with infected individuals. One potential explanation is the increased risk of reinfection or ongoing exposure to the disease. Living with infected individuals may lead to a constant threat of transmission, making it challenging for patients to fully adhere to their treatment due to the possibility of reinfection.

### Effects of non-compliance to medication among patients living with TB

Eighty five per cent of the patients with TB who showed signs of treatment non-adherence claimed to have joint discomfort or some side effects. Some anti-TB drugs, including isoniazid or pyrazinamide, may cause joint discomfort as a side effect. This finding is similar to results from a retrospective study in South Korea on side effects associated with the treatment of multidrug-resistant TB at a TB by Yang *et al*.^21^ The study found that one or more side effects were observed in 95 (37.1%) of the 256 study patients. When people experience side effects or discomfort, it may make it more difficult for patients to adhere to a treatment plan since they may find the side effects uncomfortable or unacceptable. Also, about 59% of the patients who did not take their medication as prescribed noted spinal discomfort as a side effect. Spinal TB (Pott’s disease), among other TB-related bone problems, may be a cause of back discomfort. Studies such as Meena *et al*^22^ have found in an examination of spine of a patient that there was tenderness at the upper dorsal spine that was later confirmed by MRI. Due to the pain and suffering emanating from this condition, this pain can interfere with everyday activities and general well-being, making it challenging for individuals to take their medicine as prescribed. In furtherance, a small percentage (5.1%) of non-adherent patients reported heart disorders as a complication. TB can occasionally affect the heart, resulting in conditions like pericarditis or myocarditis.^23^

## Conclusion and recommendations

### Conclusion

Smoking, eating habits and alcohol use constituted lifestyle risk factors among the study participants. The participants had a generally good degree of adherence. Adherence level was strongly influenced by factors like income, medication side effects and living with TB-infected patients. Joint and spinal discomfort, heart conditions, liver or kidney problems and other complications were mentioned by the participants.

### Recommendations

1. It is important to monitor and reduce TB treatment-related complications. Close observation, prompt action and efficient management of problems may lessen patient suffering and promote higher adherence.
2. Patients’ sense of ownership and responsibility can be cultivated by being actively involved in their treatment regimens, which may improve adherence.

### Limitations

Cause and effect relationship could not be established. Also, since this study was conducted in one district, generalising this result should be done with caution.

## Data availability statement

Data are available upon reasonable request.
